# Epidemiological Analysis of Legionella Pneumonia in Japan: A National Inpatient Database Study

**DOI:** 10.2188/jea.JE20230178

**Published:** 2024-08-05

**Authors:** Satoshi Kutsuna, Hiroyuki Ohbe, Naoki Kanda, Hiroki Matsui, Hideo Yasunaga

**Affiliations:** 1 Department of Infection Control, Graduate School of Medicine Faculty of Medicine, Osaka University, Osaka, Japan; 2 Department of Clinical Epidemiology and Health Economics, School of Public Health, The University of Tokyo, Tokyo, Japan; 3 Division of General Internal Medicine, Jichi Medical University Hospital, Tochigi, Japan

**Keywords:** Legionella pneumonia, epidemiology, mortality, hospitalization

## Abstract

**Background:**

Legionella pneumonia, a severe form of pneumonia, is caused by *Legionella* bacteria. The epidemiology of Legionnaires’ disease in Japan, including seasonal trends, risk factors for severe disease, and fatality rates, is unclear. This study examined the epidemiology of Legionella pneumonia in Japan.

**Methods:**

This retrospective cohort study included data of adult patients hospitalized for Legionella pneumonia (identified using the International Classification of Diseases, 10^th^ revision code, A481) in the Japanese Diagnosis Procedure Combination inpatient database, from April 2011 to March 2021. We performed multivariable logistic regression analysis to explore the prognostic factors of in-hospital mortality.

**Results:**

Of 7,370 enrolled hospitalized patients from 1,140 hospitals (male, 84.4%; aged >50 years, 87.9%), 469 (6.4%) died during hospitalization. The number of hospitalized patients increased yearly, from 658 in 2016 to 975 in 2020. Multivariable logistic regression analysis revealed that higher in-hospital mortality was associated with older age, male sex, lower body mass index, worsened level of consciousness, comorbidities (congestive heart failure, chronic renal diseases, and metastasis), hospitalization from November to May, and ambulance use. However, lower in-hospital mortality was associated with comorbidity (liver diseases), hospitalization after 2013, and hospitalization in hospitals with higher case volume.

**Conclusion:**

The characterized epidemiology of Legionella pneumonia in Japan revealed a high mortality rate of 6.4%. To the best of our knowledge, this is the first study to demonstrate a higher mortality rate in winter and in patients with congestive heart failure and metastasis. Further research is needed to understand the complex interplay between the prognostic factors of Legionella pneumonia.

## INTRODUCTION

Legionella pneumonia, a severe form of pneumonia, is caused by gram-negative bacteria belonging to the *Legionella* genus, with *Legionella pneumophila* as the most common species causing the disease.^1^ The *Legionella* pneumoniae infection is typically contracted by inhalation of aerosolized water droplets contaminated with the bacteria, which can be found in various water sources, such as air conditioning systems, cooling towers, and hot tubs. Although the disease is relatively rare, it poses a significant public health concern due to its potential to cause outbreaks and its high mortality rate.^2^ Several epidemiological characteristics of Legionella pneumonia have been reported globally. The risk of infection increases with older age, particularly in individuals over the age of 50 years, because they are more susceptible to the disease.^2^ Males are generally at a higher risk of infection than females, possibly due to factors like occupational exposure and lifestyle choices.^3^ The incidence of Legionella pneumonia is higher during warmer months, as the bacteria thrive in warm water environments.^3^ Individuals with weakened immune system and smokers are more vulnerable to infection.^2^ In addition, the identified prognostic factors for severe Legionella pneumonia include older age,^4^ immune system compromise,^5^ chronic lung disease,^6^ diabetes,^7^ and renal dysfunction.^8^ Understanding these global epidemiological characteristics is essential for developing effective strategies to prevent and control the spread of Legionella pneumonia in various regions. Other than patient background, prompt antimicrobial administration has been shown to improve prognosis.^9^^,^^10^ In addition, we report that patients diagnosed on the first day of admission using urinary antigen testing have a better prognosis than those diagnosed on the second or later days.^11^

In Japan, the requirement of the Infectious Diseases Control Law that healthcare providers report cases of Legionella pneumonia, has led to a better understanding of its occurrence and characteristics. However, only limited epidemiological characteristics of cases have been reported, and mortality data have been underestimated, as only deaths at the time of reporting were captured. Therefore, we aimed to examine the epidemiology of Legionella pneumonia in Japan using data from the Diagnosis Procedure Combination (DPC) database, a nationwide inpatient database that provides comprehensive information on disease occurrence and patient demographics. The purpose of this study was to analyze the detailed background of hospitalized patients with Legionella pneumonia in Japan using the DPC data and explore the prognostic factors of mortality.

## METHODS

### Data source

This retrospective cohort study used the data from a nationwide administrative inpatient database, the Japanese DPC inpatient database, which contains discharge summary and administrative claims data from more than 1,500 acute-care hospitals in Japan. These hospitals are voluntarily contributing to the database, and the data cover approximately 50% of all acute-care hospital beds in Japan.^12^ The following patient-level data for all hospitalizations are included in the database: age, sex, diagnoses recorded using the International Classification of Diseases, Tenth Revision (ICD-10) codes, daily procedures recorded, daily drug administration, admission status, and discharge summary. Previous validation study of this database showed a high specificity of 93.2% and a moderate sensitivity of 78.9% for diagnosis.^13^

### Study population

We identified adult patients hospitalized for Legionella pneumonia, defined using the primary diagnosis ICD-10 code, A481, from April 2011 to March 2021. We excluded cases of suspected Legionella pneumonia.

### Patient characteristics

We collected data on age at admission, sex, smoking history, body mass index (BMI) at admission, Japan Coma Scale score at admission, comorbidities at admission, fiscal year at admission, month of hospitalization, ambulance use, weekend admission (Saturday and Sunday), teaching hospital, tertiary emergency hospital, and hospital case volume during the study period. Age was categorized into 0–9, 10–19, 20–39, 40–59, 60–69, 70–79, 80–89, or ≥90 years; smoking history was categorized as nonsmoker, current/past smoker, and missing data were reported; BMI was categorized into <18.5, 18.5–22.9, 23–24.9, 25–29.9, or ≥30 kg/m^2^; and the Japan Coma Scale was used to describe the general level of consciousness and was categorized into alert, dizziness, somnolence, and coma. The purpose of this study was to identify risk factors for severe Legionella pneumonia, including the patient background characteristics that remain unchanged after hospitalization and intervention, by healthcare providers. Therefore, no information was collected on the details of post-hospitalization examinations or treatment and was not included in the analysis.

### Outcomes

The primary outcome was in-hospital mortality rate. Secondary outcomes were total hospitalization costs, length of hospital stay, and organ support therapy during hospitalization. Organ support therapies included intensive care unit admission, supplemental oxygen therapy, mechanical ventilation, extracorporeal membrane oxygenation, catecholamine administration, renal replacement therapy, and blood transfusion.

### Estimation of the national incidence of Legionella pneumonia in Japan

We estimated the national incidence of patients hospitalized for Legionella pneumonia in Japan based on the number of acute-care beds in all hospitals, using the Survey of the Medical Institute,^14^ and the number of patients hospitalized for Legionella pneumonia in the DPC database, stratified by acute-care bed volume categories. The estimation of the national incidence was calculated by summing the number of patients hospitalized for Legionella pneumonia in the DPC database divided by the percentage of acute care beds in all hospitals included in the DPC database stratified by acute-care bed volume categories. Because the Survey of Medical Institute data were available from fiscal year 2016, we estimated the national incidence of patients hospitalized for Legionella pneumonia from 2016 to 2020.

### Statistical analysis

Patient characteristics were compared between those who survived and those who died during hospitalization using the chi-square test for categorical variables and the *t*-test for continuous variables. We performed multi-level logistic regression analysis using generalized estimating equation approach accompanied by cluster-robust standard errors with hospitals as the clusters to investigate the association between in-hospital mortality as the dependent variable and age category, male sex, smoking history, BMI at admission, Japan Coma Scale score at admission, comorbidities at admission, fiscal year at admission, month of admission, ambulance use, weekend hospitalization, teaching hospital, tertiary emergency hospital, and hospital case volume as the independent variables.^15^ We then plotted the percentage of monthly patient admissions and the corresponding monthly in-hospital mortality from January to December.

Missing data were reported for the respective variables. All analyses were performed using STATA/SE software, version 17.0 (STATA Corp, College Station, TX, USA). All hypothesis tests were two-sided, with a significance level of 0.05.

### Sensitivity analysis

A sensitivity analysis was conducted excluding cases from 2010 and 2011, considering the possibility that cases from 2010 and 2011 may have affected the overall results due to the small number of DPC-registered hospitals. Another sensitivity analysis was conducted with multiple imputations.^16^ Some patients had missing data on smoking history and BMI. We performed multiple imputations by chained equations, creating 20 imputed datasets with all covariates and outcomes, using the “mi impute chained” command in STATA software.^17^ We combined the imputation estimates and standard errors according to Rubin’s rule.^18^ In addition to this, as noted above, we have reported better prognosis in patients tested for Legionella urinary antigen on the day of admission than in those tested on the second day or later,^11^ so we performed a sensitivity analysis focusing on patients tested on the first day of admission to appropriately estimate the risk of patient background factors.

### Ethics approval and consent to participate

This study was approved by the Institutional Review Board of the University of Tokyo (approval number, 3501-3; December 25, 2017). No identifying information of individual patients, hospitals, or physicians was allowed, and the requirement for informed consent was waived because of the anonymous nature of the data.

### Data sharing statement

The datasets analyzed in this study are not publicly available due to contracts with hospitals reporting data in the database.

## RESULTS

During the 10-year study period, 7,370 cases of Legionella pneumonia were identified in 1,140 hospitals. Based on the Survey of the Medical Institute and DPC data, the national estimates of hospitalized cases for Legionella pneumonia from 2016, 2017, 2018, 2019, and 2020 were 1,186.6, 1,230.2, 2,315.6, 2,310.2, and 1,765.7, respectively (Table 1, eTable 1, and eTable 2).

**Table 1.** The number of patients enrolled in the study between 2016 and 2020 and the national estimates of hospitalized cases for Legionella pneumonia

Number of acute-care beds, *n* (%)	Number of patients					Estimated number of patients				
	
	2016	2017	2018	2019	2020	2016	2017	2018	2019	2020
	
≤99	26	22	37	32	21	247.7	220.2	335.4	295.4	180.4
100–199	60	64	144	129	101	163.7	182.6	398.7	376.3	283.8
200–299	101	110	190	194	156	171.5	187.1	326.8	369.3	295.6
300–399	134	152	278	219	195	199.3	239.0	448.3	392.6	337.7
400–499	85	79	199	184	158	114.3	112.8	295.9	293.6	256.5
500–599	103	97	148	160	120	119.9	117.3	184.0	189.9	142.8
600–699	78	67	133	129	103	91.1	80.2	153.1	167.9	133.9
700–799	28	39	78	84	62	32.7	47.3	94.8	103.4	73.3
800–899	14	15	21	41	16	16.4	17.7	23.5	47.1	18.8
≥900	29	25	53	72	43	30.1	25.9	55.0	74.8	43.0
Total	658	670	1,281	1,244	975	1,186.6	1,230.2	2,315.6	2,310.2	1,765.7

The patients’ mean age was 67.7 years, 90.7% were over 50 years old, and 84.4% were male (Table 2). Overall, 52.3% of patients were current or past smokers. The levels of consciousness on admission for alert, dizziness, somnolence, and coma were 76.3%, 19.5%, 2.7%, and 1.5%, respectively. A total of 37.0% of the patients were transported to the hospital by ambulance.

**Table 2.** Clinical characteristics of patients enrolled in the study

Variables	Total *N* = 7,370	Survived *N* = 6,901	Died *N* = 469	*P* value
**Patient characteristics**				
Age, years, mean (SD)	67.7 (13.0)	67.0 (12.7)	78.0 (12.3)	<0.001
Age category, years, *n* (%)				
0–9	2 (0.0)	1 (0.0)	1 (0.2)	<0.001
10–19	11 (0.1)	11 (0.2)	0 (0.0)	
20–29	36 (0.5)	36 (0.5)	0 (0.0)	
30–39	127 (1.7)	125 (1.8)	2 (0.4)	
40–49	509 (6.9)	496 (7.2)	13 (2.8)	
50–59	1,294 (17.6)	1,272 (18.4)	22 (4.7)	
60–69	2,341 (31.8)	2,268 (32.9)	73 (15.6)	
70–79	1,778 (24.1)	1,658 (24.0)	120 (25.6)	
80–89	1,062 (14.4)	881 (12.8)	181 (38.6)	
>90	210 (2.8)	153 (2.2)	57 (12.2)	
Male, *n* (%)	6,217 (84.4)	5,864 (85.0)	353 (75.3)	<0.001
Smoking history, *n* (%)				
Nonsmoker	2,508 (34.0)	2,277 (33.0)	231 (49.3)	<0.001
Current/past smoker	3,855 (52.3)	3,691 (53.5)	164 (35.0)	
Data missing	1,007 (13.7)	933 (13.5)	74 (15.8)	
Body mass index at admission, kg/m^2^, *n* (%)				
<18.5	636 (8.6)	535 (7.8)	101 (21.5)	<0.001
18.5–24.9	4,079 (55.3)	3,880 (56.2)	199 (42.4)	
25.0–29.9	1,489 (20.2)	1,431 (20.7)	58 (12.4)	
≥30.0	325 (4.4)	311 (4.5)	14 (3.0)	
Data missing	841 (11.4)	744 (10.8)	97 (20.7)	
Japan Coma Scale at admission, *n* (%)				
Alert	5,622 (76.3)	5,375 (77.9)	247 (52.7)	<0.001
Dizziness	1,436 (19.5)	1,297 (18.8)	139 (29.6)	
Somnolence	199 (2.7)	153 (2.2)	46 (9.8)	
Coma	113 (1.5)	76 (1.1)	37 (7.9)	
Comorbidities at admission, *n* (%)				
Myocardial infarction	111 (1.5)	104 (1.5)	7 (1.5)	0.98
Congestive heart failure	676 (9.2)	583 (8.4)	93 (19.8)	<0.001
Peripheral vascular diseases	77 (1.0)	70 (1.0)	7 (1.5)	0.32
Cerebral vascular diseases	379 (5.1)	344 (5.0)	35 (7.5)	0.019
Dementia	229 (3.1)	201 (2.9)	28 (6.0)	<0.001
Chronic pulmonary diseases	581 (7.9)	540 (7.8)	41 (8.7)	0.48
Connective tissue diseases	191 (2.6)	179 (2.6)	12 (2.6)	0.96
Peptic ulcer diseases	234 (3.2)	225 (3.3)	9 (1.9)	0.11
Liver diseases	583 (7.9)	569 (8.2)	14 (3.0)	<0.001
Diabetes mellitus	1,762 (23.9)	1,673 (24.2)	89 (19.0)	0.010
Chronic renal diseases	341 (4.6)	292 (4.2)	49 (10.4)	<0.001
Malignancy	335 (4.5)	305 (4.4)	30 (6.4)	0.047
Metastasis	31 (0.4)	18 (0.3)	13 (2.8)	<0.001
HIV	8 (0.1)	7 (0.1)	1 (0.2)	0.48
Fiscal year of hospitalization, *n* (%)				
2010–2012	864 (11.7)	775 (11.2)	89 (19.0)	<0.001
2013–2014	1,006 (13.6)	926 (13.4)	80 (17.1)	
2015–2016	1,300 (17.6)	1,225 (17.8)	75 (16.0)	
2017–2018	1,971 (26.7)	1,869 (27.1)	102 (21.7)	
2019–2020	2,229 (30.2)	2,106 (30.5)	123 (26.2)	
Month of hospitalization, *n* (%)				
January	436 (5.9)	382 (5.5)	54 (11.5)	<0.001
February	357 (4.8)	323 (4.7)	34 (7.2)	
March	361 (4.9)	331 (4.8)	30 (6.4)	
April	314 (4.3)	282 (4.1)	32 (6.8)	
May	543 (7.4)	505 (7.3)	38 (8.1)	
June	803 (10.9)	774 (11.2)	29 (6.2)	
July	1,108 (15.0)	1,067 (15.5)	41 (8.7)	
August	750 (10.2)	713 (10.3)	37 (7.9)	
September	854 (11.6)	823 (11.9)	31 (6.6)	
October	780 (10.6)	733 (10.6)	47 (10.0)	
November	605 (8.2)	553 (8.0)	52 (11.1)	
December	459 (6.2)	415 (6.0)	44 (9.4)	
Weekend admission, *n* (%)	1,468 (19.9)	1,367 (19.8)	101 (21.5)	0.37
Ambulance use, *n* (%)	2,730 (37.0)	2,430 (35.2)	300 (64.0)	<0.001
Teaching hospital, *n* (%)	6,878 (93.3)	6,436 (93.3)	442 (94.2)	0.41
Tertiary emergency hospital, *n* (%)	2,769 (37.6)	2,586 (37.5)	183 (39.0)	0.50
Hospital case volume, mean (SD)	3.0 (2.8)	3.1 (2.8)	2.7 (2.0)	0.005
**Outcomes**				
In-hospital mortality (%)	469 (6.4)	—	469 (100.0)	<0.001
Total hospitalization cost, thousand Japanese yen, median (IQR)	571 (406–907)	562 (406–857)	995 (410–2,217)	<0.001
Length of hospital stay, days, median (IQR)	13.0 (9.0–20.0)	13.0 (9.0–20.0)	13.0 (5.0–28.0)	0.046
Organ support therapies, *n* (%)				
ICU/HCU admission	1,433 (19.4)	1,229 (17.8)	204 (43.5)	<0.001
Supplemental oxygen	4,408 (59.8)	4,070 (59.0)	338 (72.1)	<0.001
Mechanical ventilation	869 (11.8)	610 (8.8)	259 (55.2)	<0.001
ECMO	48 (0.7)	32 (0.5)	16 (3.4)	<0.001
Catecholamine	704 (9.6)	473 (6.9)	231 (49.3)	<0.001
Renal replacement therapy	350 (4.7)	252 (3.7)	98 (20.9)	<0.001
Blood transfusion	456 (6.2)	312 (4.5)	144 (30.7)	<0.001

HCU, high care unit; HIV, human immunodeficiency virus; ICU, intensive care unit; IQR, interquartile range; ECMO, extracorporeal membrane oxygenation; SD, standard deviation.

Of the 7,370 cases, 469 (6.4%) died during hospitalization. The median total hospitalization cost was 571,000 Japanese yen, and the median length of stay was 13.0 days. A total of 19.4% of the patients were admitted to the intensive care unit or high-dependency care unit. Supplemental oxygen, mechanical ventilation, extracorporeal membrane oxygenation, catecholamine use, renal replacement therapy, and blood transfusion were performed in 59.8%, 11.8%, 0.7%, 9.6%, 4.7%, and 6.2% of the patients, respectively.

After adjusting for patient background in the multi-level analysis, older age, male sex, lower BMI, worsened level of consciousness at admission, comorbidities (congestive heart failure, chronic renal diseases, and metastasis), month (November to May), and ambulance use were associated with higher in-hospital mortality. In contrast, comorbidity (liver diseases), hospitalization after 2013, and admission to hospitals with higher hospital case volume were associated with lower in-hospital mortality (Table 3). The results of sensitivity analyses were similar with those in the main analysis (eTable 3 and eTable 4). A sensitivity analysis restricted to only those patients who had Legionella urinary antigen testing on the day of admission was also performed, but the results did not differ significantly (eTable 5).

**Table 3.** Analysis of mortality risk factors after adjusting for patient background in the multivariable logistic regression model

Variables	Odds ratio (95% Cis)	*P* value
Age category, years		
0–9	—	—
10–19	—	—
20–29	—	—
30–39	1.00 (0.29–3.48)	1.00
40–49	1.67 (0.82–3.40)	0.16
50–59	Reference	
60–69	1.97 (1.21–3.22)	0.007
70–79	3.85 (2.39–6.19)	<0.001
80–89	8.15 (5.02–13.21)	<0.001
>90	13.29 (7.46–23.68)	<0.001
Male	1.39 (1.05–1.84)	0.023
Smoking history		
Nonsmoker	Reference	
Current/past smoker	0.80 (0.62–1.03)	0.088
Data missing	0.85 (0.62–1.16)	0.30
Body mass index at admission, kg/m^2^		
<18.5	2.10 (1.57–2.81)	<0.001
18.5–24.9	Reference	
25.0–29.9	0.98 (0.71–1.35)	0.90
≥30.0	1.52 (0.84–2.76)	0.17
Data missing	2.03 (1.53–2.69)	<0.001
Japan Coma Scale at admission		
Alert	Reference	
Dizziness	1.41 (1.11–1.80)	0.006
Somnolence	2.68 (1.79–4.02)	<0.001
Coma	5.00 (3.07–8.15)	<0.001
Comorbidities at admission		
Myocardial infarction	0.68 (0.29–1.57)	0.36
Congestive heart failure	1.45 (1.10–1.91)	0.009
Peripheral vascular diseases	0.97 (0.42–2.26)	0.95
Cerebral vascular diseases	0.86 (0.58–1.27)	0.44
Dementia	0.66 (0.42–1.04)	0.074
Chronic pulmonary diseases	1.02 (0.71–1.47)	0.92
Connective tissue diseases	0.73 (0.38–1.39)	0.33
Peptic ulcer diseases	0.57 (0.28–1.17)	0.13
Liver diseases	0.55 (0.32–0.97)	0.038
Diabetes mellitus	0.82 (0.63–1.06)	0.13
Chronic renal diseases	1.97 (1.37–2.82)	<0.001
Malignancy	0.85 (0.54–1.34)	0.48
Metastasis	8.82 (3.81–20.43)	<0.001
HIV	2.81 (0.29–27.58)	0.38
Fiscal year of hospitalization		
2010–2012	Reference	
2013–2014	0.65 (0.46–0.93)	0.018
2015–2016	0.53 (0.37–0.75)	<0.001
2017–2018	0.47 (0.34–0.65)	<0.001
2019–2020	0.50 (0.36–0.69)	<0.001
Month of hospitalization		
January	2.15 (1.36–3.42)	0.001
February	1.63 (0.97–2.73)	0.063
March	1.81 (1.07–3.05)	0.026
April	2.13 (1.27–3.57)	0.004
May	2.01 (1.24–3.27)	0.005
June	0.95 (0.57–1.58)	0.84
July	Reference	
August	1.23 (0.76–2.00)	0.39
September	0.94 (0.57–1.55)	0.82
October	1.47 (0.93–2.33)	0.096
November	1.70 (1.08–2.69)	0.023
December	1.96 (1.22–3.14)	0.005
Weekend	0.94 (0.73–1.21)	0.64
Ambulance use	2.30 (1.85–2.87)	<0.001
Teaching hospital	1.08 (0.68–1.70)	0.75
Tertiary emergency hospital	1.00 (0.79–1.26)	0.98
Hospital case volume	0.95 (0.90–1.00)	0.045

HIV, human immunodeficiency virus.

**Figure 1.**
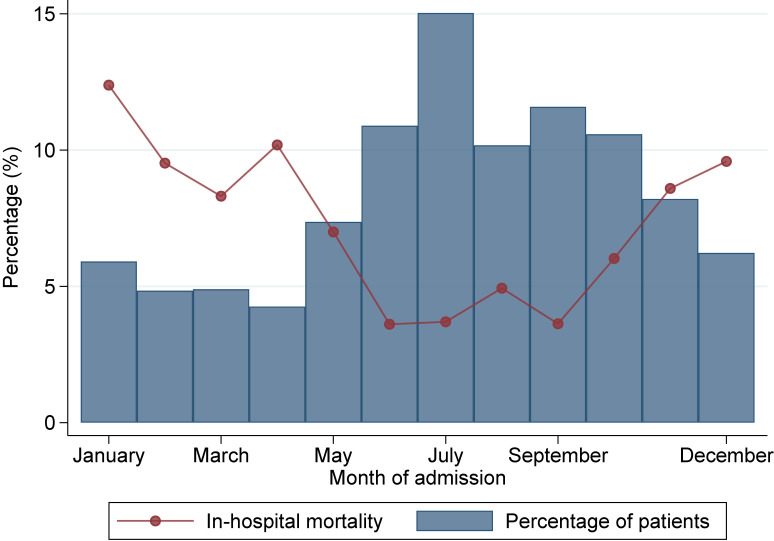
The percentage of monthly patient admissions and corresponding monthly in-hospital mortality from January to December

The percentage of monthly patient admissions and corresponding monthly in-hospital mortality from January to December are shown in Figure 1. During the relatively warmer months (May to October), there was a higher proportion of patients and lower in-hospital mortality than during the colder months (November to April). The month with the lowest in-hospital mortality was June, with a rate of 3.6%, while the month with the highest rate was in January, at 12.4%.

## DISCUSSION

In this study, we characterized the epidemiology of Legionella pneumonia in Japan by analyzing DPC data from April 2011 to March 2021. Our results show that the mortality rate of hospitalized patients with Legionella pneumonia in Japan is as high as 6.4% and that the mortality rate is higher in winter, in addition to the previously known prognostic factor indicating a higher rate in summer. To the best of our knowledge, this is the first study to demonstrate that congestive heart failure and metastasis are risk factors for severe disease.

Our study revealed the mortality rates of hospitalized patients with Legionella pneumonia in Japan. A compilation of 10 years of data from 2007 on Legionella pneumonia cases reported under the Infectious Disease Control Law showed a mortality rate of 1.9% at the time of reporting.^19^ This reporting may have reflected an underestimation because deaths after reporting were not captured. In recent years, the number of cases reported has been increasing, especially since rapid tests, such as the urine antigen test and the loop-mediated isothermal amplification (LAMP) method, have made it possible to quickly diagnose the disease. We have previously reported that early diagnosis of Legionella pneumonia correlates with prognosis.^11^ Although we were assessing the risk of *Legionella* pneumonia patients at the time of admission, the patients included in this study were those with *Legionella* pneumonia who were given a DPC disease title at the time of discharge, so there is a gap in the timing of diagnosis. To fill this gap, we also performed a sensitivity analysis limited to patients who had Legionella urine antigen testing on the day of admission, but there was no significant difference in the results.

Our findings revealed that the incidence of Legionella pneumonia was higher during the summer months, whereas the mortality rate was higher during the winter. This seasonal pattern can be attributed to various factors, as supported by existing literature. The higher incidence of Legionella pneumonia in the summer months can be explained by the increased use of air conditioning systems and cooling towers, which provide suitable environments for the growth and transmission of Legionella bacteria.^20^ Higher temperatures and humidity during the summer can also promote bacterial growth, increasing the risk of infection.^21^ Additionally, increased travel and tourism during the summer can contribute to contaminated water sources’ exposure in hotels, resorts, and other accommodation facilities.^22^ Conversely, the higher mortality rate observed during the winter season might be related to several factors. First, patients with Legionella pneumonia might develop more severe symptoms and complications in the winter, due to co-infection with other respiratory pathogens, such as influenza virus.^8^ Second, patients’ immune system might be more vulnerable during the colder months, leading to a higher risk of severe outcomes.^23^ Lastly, the diagnosis of Legionella pneumonia might be delayed in the winter due to the overlap of symptoms with other respiratory illnesses, which could result in late treatment and increased mortality.^24^ While various respiratory infections such as influenza are prevalent during the winter months, we must also properly diagnose and promptly treat Legionella pneumonia.

In this study, we estimated the total number of hospitalized patients with Legionella pneumonia in Japan based on the number of cases registered in the DPC and the number of hospital beds registered in the Survey of Medical Institutions. Our study estimated that 1,186.6, 1,230.2, 2,315.6, 2,310.2, and 1,765.7 cases of Legionella pneumonia were hospitalized in 2016, 2017, 2018, 2019, and 2020, respectively, throughout Japan. The number of Legionella pneumonia cases reported to the National Institute of Infectious Diseases was 1,602, 1,733, 2,142, 2,316, and 2,059 in 2016, 2017, 2018, 2019, and 2020, respectively. Although not all notified patients were likely to be hospitalized, our estimates did not deviate significantly from the number of notified reports, and this method is considered useful for estimating the actual number of infected patients.

The present study has some limitations. First, although we included cases registered as Legionella pneumonia in the DPC database, we cannot guarantee that diagnoses were based on ideal methods. Although Legionella pneumonia occurrence is thought to have increased in recent years with the availability of urine antigen tests and the LAMP method, these tests are considered to have a high degree of specificity; therefore, the reliability of the reporting of the disease in the DPC is considered reasonably high. When a disease is recorded using an ICD-10 code in DPC, the specificity is very high and the sensitivity is moderate; therefore, it is likely that the disease is truly Legionella pneumonia.^13^ Furthermore, it has been reported that 75% of hospitalized patients recorded in the DPC as Legionella pneumonia were tested for *Legionella pneumophilla* on the first day of hospitalization.^11^ Of the 7,370 patients included in our study, 5,746 (78.0%) were tested for *Legionella pneumophilla* on the day of admission while 6,768 (91.8%) were tested during hospitalization. This suggests that the diagnosis of many of the cases included in our study were based on testing. Second, the DPC database included only hospitalized patients, potentially underrepresenting milder cases of Legionella pneumonia that did not require hospitalization. This could skew the results toward more severe cases, thus affecting the generalizability of the findings. Third, this study focused on patient background only, and did not include analysis of examination or treatment details. It is possible that new testing techniques or antimicrobial approvals may have affected disease outcomes. In conclusion, we analyzed the Japanese DPC data to determine the epidemiological characteristics of hospitalized patients with Legionella pneumonia. Further research is needed to understand the complex interplay between the prognostic factors of Legionella pneumonia.

## SUPPLEMENTARY MATERIAL

eTable 1. Number of acute-care beds in all hospitals from the Survey of the Medical Institute

eTable 2. Number of acute-care beds in the DPC database and their percentage of all hospitals

eTable 3. Analysis of mortality risk factors after adjusting for patient background in the Multiple imputation

eTable 4. Sensitivity analysis excluding 2010–2012

eTable 5. Sensitivity analysis restricted to only patients who had Legionella urinary antigen testing on the day of admission
