# Supplementary material for: Epidemiological Analysis of Legionella Pneumonia in Japan: A National Inpatient Database Study
Source: J Epidemiol. 2024 Aug 5;34(8):365–71. doi: 10.2188/jea.JE20230178 (PMC11230878; doi:10.2188/jea.JE20230178)
Supplement: Supplementary file 1 [file je-34-365-s001.pdf]

eTable 1. Number of acute-care beds in all hospitals from the Survey of the Medical Institute

Category of acute-care bed volume	Number of acute-care beds in all hospitals				
	2016	2017	2018	2019	2020
≤99	145,201	145,009	139,843	133,976	131,273
100–199	122,366	120,697	116,234	111,911	108,639
200–299	85,471	86,096	85,127	83,892	78,738
300–399	100,041	93,560	91,336	90,835	89,639
400–499	68,511	69,777	66,564	63,403	60,573
500–599	57,427	56,421	58,280	61,497	57,111
600–699	46,297	46,973	44,462	39,064	39,659
700–799	25,883	25,384	25,540	23,956	24,752
800–899	17,612	16,763	15,878	19,202	16,714
≥900	30,167	30,213	29,804	29,188	27,748
Total	698,976	690,893	673,068	656,924	634,846

eTable 2. Number of acute-care beds in the DPC database and their percentage of all hospitals

Category of acute-care bed volume	Number of acute-care beds in the DPC database					Percentage of acute-care beds in the DPC database				
	2016	2017	2018	2019	2020	2016	2017	2018	2019	2020
≤99	15,241	14,487	15,425	14,513	15,281	10.5%	10.0%	11.0%	10.8%	11.6%
100–199	44,841	42,301	41,978	38,364	38,667	36.6%	35.0%	36.1%	34.3%	35.6%
200–299	50,326	50,608	49,491	44,073	41,558	58.9%	58.8%	58.1%	52.5%	52.8%
300–399	67,267	59,514	56,634	50,669	51,765	67.2%	63.6%	62.0%	55.8%	57.7%
400–499	50,964	48,853	44,771	39,732	37,307	74.4%	70.0%	67.3%	62.7%	61.6%
500–599	49,323	46,665	46,883	51,824	48,000	85.9%	82.7%	80.4%	84.3%	84.0%
600–699	39,657	39,248	38,634	30,009	30,503	85.7%	83.6%	86.9%	76.8%	76.9%
700–799	22,185	20,912	21,016	19,467	20,950	85.7%	82.4%	82.3%	81.3%	84.6%
800–899	15,056	14,236	14,166	16,718	14,243	85.5%	84.9%	89.2%	87.1%	85.2%
≥900	29,079	29,125	28,716	28,100	27,748	96.4%	96.4%	96.3%	96.3%	100.0%
Total	383,939	365,949	357,714	333,469	326,022	54.9%	53.0%	53.1%	50.8%	51.4%

DPC, Diagnosis Procedure Combination.

eTable 3. Analysis of mortality risk factors after adjusting for patient background in the Multiple imputation

Variables	Odds ratio (95% CI)	*P* value
Age category, years		
0–9	–	–
10–19	–	–
20–29	–	–
30–39	1.02 (0.30–3.54)	0.97
40–49	1.64 (0.81–3.34)	0.17
50–59	Reference	
60–69	1.92 (1.18–3.14)	0.009
70–79	3.79 (2.36–6.09)	<0.001
80–89	8.07 (4.97–13.13)	<0.001
>90	13.11 (7.34–23.43)	<0.001
Male	1.36 (1.03–1.80)	0.033
Smoking history		
Nonsmoker	Reference	
Current/past smoker	0.85 (0.66–1.08)	0.18
Body mass index at admission, kg/m^2		
<18.5	1.87 (1.41–2.49)	<0.001
18.5–24.9	Reference	
25.0–29.9	0.94 (0.68–1.29)	0.68
≥30.0	1.32 (0.72–2.43)	0.37
Japan Coma Scale at admission		
Alert	Reference	
Dizziness	1.44 (1.13–1.84)	0.003
Somnolence	2.73 (1.82–4.09)	<0.001
Coma	5.02 (3.08–8.17)	<0.001
Comorbidities at admission		
Myocardial infarction	0.65 (0.28–1.51)	0.32
Congestive heart failure	1.45 (1.10–1.91)	0.008
Peripheral vascular diseases	1.00 (0.43–2.33)	0.99
Cerebral vascular diseases	0.84 (0.57–1.25)	0.40
Dementia	0.67 (0.43–1.06)	0.084
Chronic pulmonary diseases	1.01 (0.70–1.46)	0.95
Connective tissue diseases	0.73 (0.38–1.40)	0.35
Peptic ulcer diseases	0.56 (0.27–1.14)	0.11

Liver diseases	0.55 (0.32–0.97)	0.038
Diabetes mellitus	0.81 (0.63–1.05)	0.12
Chronic renal diseases	1.93 (1.35–2.77)	<0.001
Malignancy	0.82 (0.52–1.29)	0.39
Metastasis	8.69 (3.75–20.11)	<0.001
HIV	2.59 (0.26–25.45)	0.41
Fiscal year of hospitalization		
2010–2012	Reference	
2013–2014	0.65 (0.46–0.93)	0.017
2015–2016	0.51 (0.36–0.73)	<0.001
2017–2018	0.46 (0.33–0.64)	<0.001
2019–2020	0.48 (0.35–0.67)	<0.001
Month of hospitalization		
January	2.18 (1.38–3.46)	0.001
February	1.64 (0.98–2.74)	0.06
March	1.81 (1.07–3.04)	0.026
April	2.14 (1.27–3.60)	0.004
May	2.02 (1.24–3.27)	0.004
June	0.95 (0.57–1.58)	0.84
July	Reference	
August	1.23 (0.76–1.99)	0.41
September	0.94 (0.57–1.55)	0.81
October	1.48 (0.94–2.33)	0.093
November	1.74 (1.10–2.75)	0.017
December	1.96 (1.22–3.14)	0.005
Weekend	0.95 (0.74–1.22)	0.67
Ambulance use	2.35 (1.89–2.93)	<0.001
Teaching hospital	1.13 (0.71–1.78)	0.61
Tertiary emergency hospital	0.99 (0.79–1.25)	0.95
Hospital case volume	0.95 (0.90–1.00)	0.057

CI, confidence interval; HIV, human immunodeficiency virus.

eTable 4. Sensitivity analysis excluding 2010–2012

Variables	Odds ratio (95% CI)	*P* value
Age category, years		
0–9	–	–
10–19	–	–
20–29	–	–
30–39	1.14 (0.31–4.15)	0.84
40–49	1.43 (0.64–3.17)	0.39
50–59	Reference	
60–69	1.63 (0.94–2.83)	0.080
70–79	3.56 (2.11–6.01)	<0.001
80–89	7.68 (4.51–13.08)	<0.001
>90	13.38 (7.14–25.09)	<0.001
Male	1.32 (0.97–1.79)	0.082
Smoking history		
Nonsmoker	Reference	
Current/past smoker	0.80 (0.61–1.06)	0.13
Data missing	0.87 (0.62–1.24)	0.45
Body mass index at admission, kg/m^2		
<18.5	2.08 (1.50–2.87)	<0.001
18.5–24.9	Reference	
25.0–29.9	1.11 (0.79–1.57)	0.55
≥30.0	1.63 (0.86–3.08)	0.14
Data missing	2.15 (1.57–2.94)	<0.001
Japan Coma Scale at admission		
Alert	Reference	
Dizziness	1.38 (1.05–1.82)	0.02
Somnolence	2.41 (1.53–3.78)	<0.001
Coma	5.13 (3.03–8.68)	<0.001
Comorbidities at admission		
Myocardial infarction	0.60 (0.23–1.60)	0.31
Congestive heart failure	1.42 (1.05–1.93)	0.024
Peripheral vascular diseases	1.00 (0.40–2.49)	1.00
Cerebral vascular diseases	0.86 (0.56–1.33)	0.50
Dementia	0.65 (0.40–1.06)	0.082
Chronic pulmonary diseases	1.10 (0.73–1.65)	0.65
Connective tissue diseases	0.71 (0.35–1.44)	0.34

Peptic ulcer diseases	0.52 (0.23–1.19)	0.12
Liver diseases	0.47 (0.24–0.91)	0.024
Diabetes mellitus	0.73 (0.55–0.98)	0.035
Chronic renal diseases	1.85 (1.25–2.75)	0.002
Malignancy	0.91 (0.55–1.50)	0.71
Metastasis	8.49 (3.42–21.04)	<0.001
HIV	3.39 (0.32–35.38)	0.31
Fiscal year of hospitalization		
2013–2014	Reference	
2015–2016	0.83 (0.58–1.19)	0.31
2017–2018	0.73 (0.52–1.02)	0.066
2019–2020	0.77 (0.55–1.07)	0.12
Month of hospitalization		
January	2.32 (1.40–3.84)	0.001
February	1.80 (1.03–3.15)	0.039
March	1.85 (1.05–3.26)	0.033
April	1.91 (1.08–3.39)	0.027
May	1.92 (1.12–3.30)	0.018
June	0.87 (0.49–1.55)	0.64
July	Reference	
August	1.24 (0.74–2.09)	0.42
September	0.93 (0.54–1.60)	0.79
October	1.34 (0.81–2.23)	0.26
November	2.03 (1.23–3.33)	0.005
December	2.04 (1.21–3.42)	0.007
Weekend	0.86 (0.65–1.14)	0.29
Ambulance use	2.22 (1.74–2.84)	<0.001
Teaching hospital	0.89 (0.56–1.43)	0.64
Tertiary emergency hospital	1.06 (0.82–1.37)	0.65
Hospital case volume	0.95 (0.91–1.01)	0.077

CI, confidence interval; HIV, human immunodeficiency virus.

eTable 5. Sensitivity analysis restricted to only patients who had Legionella urinary antigen testing on the day of admission

Variables	Odds ratio (95% CI)	*P* value
Age category, years		
0–9	–	–
10–19	–	–
20–29	–	–
30–39	–	–
40–49	1.60 (0.73–3.49)	0.24
50–59	Reference	
60–69	1.71 (0.99–2.95)	0.052
70–79	3.02 (1.78–5.11)	<0.001
80–89	6.46 (3.78–11.01)	<0.001
>90	11.85 (6.20–22.68)	<0.001
Male	1.32 (0.95–1.84)	0.095
Smoking history		
Nonsmoker	Reference	
Current/past smoker	0.76 (0.56–1.02)	0.07
Data missing	0.80 (0.55–1.16)	0.24
Body mass index at admission, kg/m^2		
<18.5	2.29 (1.63–3.22)	<0.001
18.5–24.9	Reference	
25.0–29.9	0.81 (0.55–1.20)	0.29
≥30.0	1.10 (0.51–2.37)	0.81
Data missing	2.17 (1.57–3.00)	<0.001
Japan Coma Scale at admission		
Alert	Reference	
Dizziness	1.44 (1.08–1.91)	0.012
Somnolence	3.27 (2.08–5.13)	<0.001
Coma	4.90 (2.79–8.59)	<0.001
Comorbidities at admission		
Myocardial infarction	0.77 (0.30–1.99)	0.59
Congestive heart failure	1.44 (1.04–1.98)	0.028
Peripheral vascular diseases	0.76 (0.26–2.27)	0.63
Cerebral vascular diseases	0.82 (0.50–1.33)	0.42
Dementia	0.47 (0.26–0.83)	0.009
Chronic pulmonary diseases	0.77 (0.49–1.23)	0.28

Connective tissue diseases	0.65 (0.30–1.41)	0.28
Peptic ulcer diseases	0.66 (0.30–1.43)	0.29
Liver diseases	0.47 (0.24–0.95)	0.036
Diabetes mellitus	0.83 (0.61–1.12)	0.215
Chronic renal diseases	1.62 (1.04–2.52)	0.034
Malignancy	0.71 (0.40–1.26)	0.25
Metastasis	6.89 (2.36–20.11)	<0.001
HIV	4.10 (0.35–48.78)	0.26
Fiscal year of hospitalization		
2010–2012	Reference	
2013–2014	0.50 (0.32–0.77)	0.002
2015–2016	0.46 (0.31–0.71)	<0.001
2017–2018	0.45 (0.30–0.66)	<0.001
2019–2020	0.45 (0.31–0.66)	<0.001
Month of hospitalization		
January	2.49 (1.43–4.34)	0.001
February	1.78 (0.97–3.27)	0.062
March	1.98 (1.07–3.67)	0.03
April	2.42 (1.32–4.43)	0.004
May	1.84 (1.01–3.36)	0.046
June	1.06 (0.58–1.92)	0.86
July	Reference	
August	1.56 (0.89–2.73)	0.12
September	0.85 (0.46–1.58)	0.61
October	1.48 (0.86–2.54)	0.16
November	1.68 (0.96–2.95)	0.069
December	2.46 (1.42–4.28)	0.001
Weekend	0.89 (0.66–1.21)	0.467
Ambulance use	2.90 (2.22–3.77)	<0.001
Teaching hospital	0.88 (0.51–1.54)	0.66
Tertiary emergency hospital	1.01 (0.77–1.32)	0.95
Hospital case volume	0.95 (0.90–1.01)	0.097

CI, confidence interval; HIV, human immunodeficiency virus.
